# Feasibility and Safety of Virtual Reality-Based Online Group Discussions Among Nursing Students: A Cross-Sectional Study

**DOI:** 10.7759/cureus.78089

**Published:** 2025-01-27

**Authors:** Hiroki Funao, Motomu Shimaoka, Jun Kako

**Affiliations:** 1 Department of Nursing, Mie University Graduate School of Medicine, Tsu, JPN; 2 Department of Molecular Pathobiology and Cell Adhesion Biology, Mie University Graduate School of Medicine, Tsu, JPN

**Keywords:** avatar, cognitive load, group discussion, nursing education, psychological safety, teamwork, video conferencing, virtual reality (vr), vr conferencing

## Abstract

Objective

Virtual reality (VR) has emerged as a potential tool for enhancing learning experiences in various fields, including nursing education. However, its feasibility, safety, and impact on online group discussions remain underexplored. This study aimed to assess the feasibility and safety of VR conferencing in basic education courses for nursing students.

Methods

This cross-sectional study was conducted with third-year nursing students at Mie University, Tsu, Japan. Participants were assigned non-randomly to either a VR discussion group using MetaQuest 3 and Horizon Workrooms or a video discussion group using Zoom meetings. The groups discussed challenges in the daily lives of patients with chronic shoulder pain and completed tasks, such as listing and ranking challenges. After the discussions, data on discussion drop rates, levels of understanding, teamwork, psychological safety, cybersickness, cognitive load, and impressions of VR and avatar usage were collected. All variables are summarized by the group as means and standard deviations, medians and interquartile ranges, or frequencies and percentages, as appropriate.

Results

A total of 36 nursing students participated in this study (VR group: n=17; video group: n=19). All participants completed the discussions without dropping out or experiencing cybersickness. The average discussion time was comparable between the groups (approximately 10 minutes). Both groups demonstrated an equivalent understanding of the topic. Both discussion methods had positive effects on teamwork and psychological safety. Cognitive load differences were mixed, with VR reducing some elements of task-related stress (e.g., understanding the overall discussion) but showing higher demands in others (e.g., the design for learning tasks). Participants in the VR group reported a high intention to continue using VR and noted that avatar customization (e.g., clothing and hairstyles) influenced the discussions.

Conclusions

Both VR and video groups engaged in discussions of similar quality and completed the discussion. As none of the participants had cybersickness or dropped out of the discussions, it was thought that the VR discussions were feasible. Carefully selecting discussion themes, evaluation items, and avatar conditions, as well as verifying the effectiveness of online discussion methods, are necessary.

## Introduction

Telehealth, the delivery of healthcare services over long distances using information and communication technologies, has spread rapidly worldwide, especially since COVID-19. One of the key elements in providing quality telemedicine services is online communication skills, which must be developed through basic professional training [1]. The main communication tool used in telemedicine is videoconferencing, wherein users share video via personal computers or tablets, with online communication skills training centered on this method [1]. However, this method presents several problems, collectively referred to as “Zoom fatigue.” This requires increased focus and effort compared with face-to-face communication, leading to increased cognitive load and poor-quality communication due to the lack of non-verbal cues, such as gestures, and a reduced sense of interpersonal distance [2]. In the medical field, multidisciplinary team meetings are essential for ensuring smooth communication, potentially expanding the range of examination and treatment options for patients, and improving one- and five-year survival rates compared to cases where such multidisciplinary team meetings are not conducted [3]. However, during Zoom meetings across Antimicrobial Stewardship (AMS) teams under COVID-19 hospital restrictions, challenges were reported to hinder trust-building within teams and reduce overall performance across teams [4].

Virtual reality (VR) has recently been used as an alternative to video conferencing. In VR conferencing, users enter a three-dimensional virtual conference room and communicate through avatars. These avatars typically reflect the user’s voice, sound direction, facial orientation, and arm movement, creating a sense of face-to-face communication [5]. This suggests that VR conferencing renders quality communication comparable to face-to-face communication [6].

Despite its potential, VR conferencing technology has yet to be widely adopted in the healthcare sector. Given that nurses play a central role in medical communication [7], we believe that acquiring the skills to utilize VR conferencing is essential for nurses. Furthermore, nursing students, as future healthcare professionals, will increasingly be expected to develop these skills.

In the field of education, VR conferencing has been shown to improve medical students’ understanding of lesson content more effectively than video conferencing [8]. Furthermore, in higher education, VR environments have been reported to enhance communication and foster a sense of community in group work, although these studies are outside the medical field, including nursing [9]. However, no studies have compared VR and video conferencing in nursing education.

Therefore, this study aimed to assess the feasibility and safety of VR conferencing for nursing students. The results of this study may be useful in forming a basis for incorporating VR conferencing into future basic nursing education programs.

## Materials and methods

Study design

This cross-sectional study compared the effects of two online group discussion methods on nursing students. This study was approved by the Institutional Review Board of the Clinical Research Ethics Review Committee at Mie University Hospital (approval no.: U2024-009). All participants provided written informed consent before data collection. The reporting followed the Strengthening the Reporting of Observational Studies in Epidemiology guidelines [10].

Setting

This single-center study was conducted at Mie University in Tsu, Japan, with data collected between September 12, 2024 and October 21, 2024.

Participants

Third-year students at the School of Nursing, Faculty of Medicine, Mie University, were invited to participate in the study, as they had prior experience with group discussion activities and had learned basic medical communication skills. In addition, a lack of history of adverse events related to VR device use was considered an eligibility criterion. To invite the students to participate in the study, an e-mail was sent to all the students explaining the study. Participants selected their preferred dates for data collection from a pre-set schedule provided in the e-mail. Based on their selected dates, they were assigned to one of two groups using different online discussion methods: the VR discussion group (VR group) and the video discussion group (video group). The number of participants per group was set at three to five, as this was considered an optimal size to improve team performance and learning [11]. Since this was a feasibility study, the sample size was not calculated. It was assumed that 20 students would participate in each group, considering the scale at which the study could be implemented.

Online group discussion

VR Group

**Figure 1:**
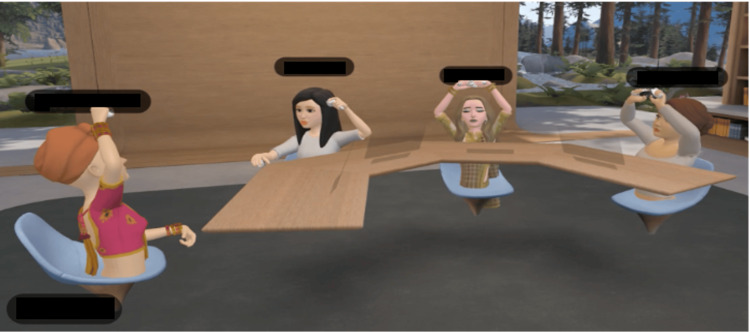
Discussions in Meta Horizon Workrooms - VR discussion group The participants in the VR group joined the discussions at Horizon Workrooms, which provide immersive VR spaces that enable users to communicate remotely through avatars. Image credit: Hiroki Funao This figure is an original creation by the author, Hiroki Funao. The image was created by capturing the application screen using the screenshot function on a MacOS PC. VR: virtual reality

**Figure 2:**
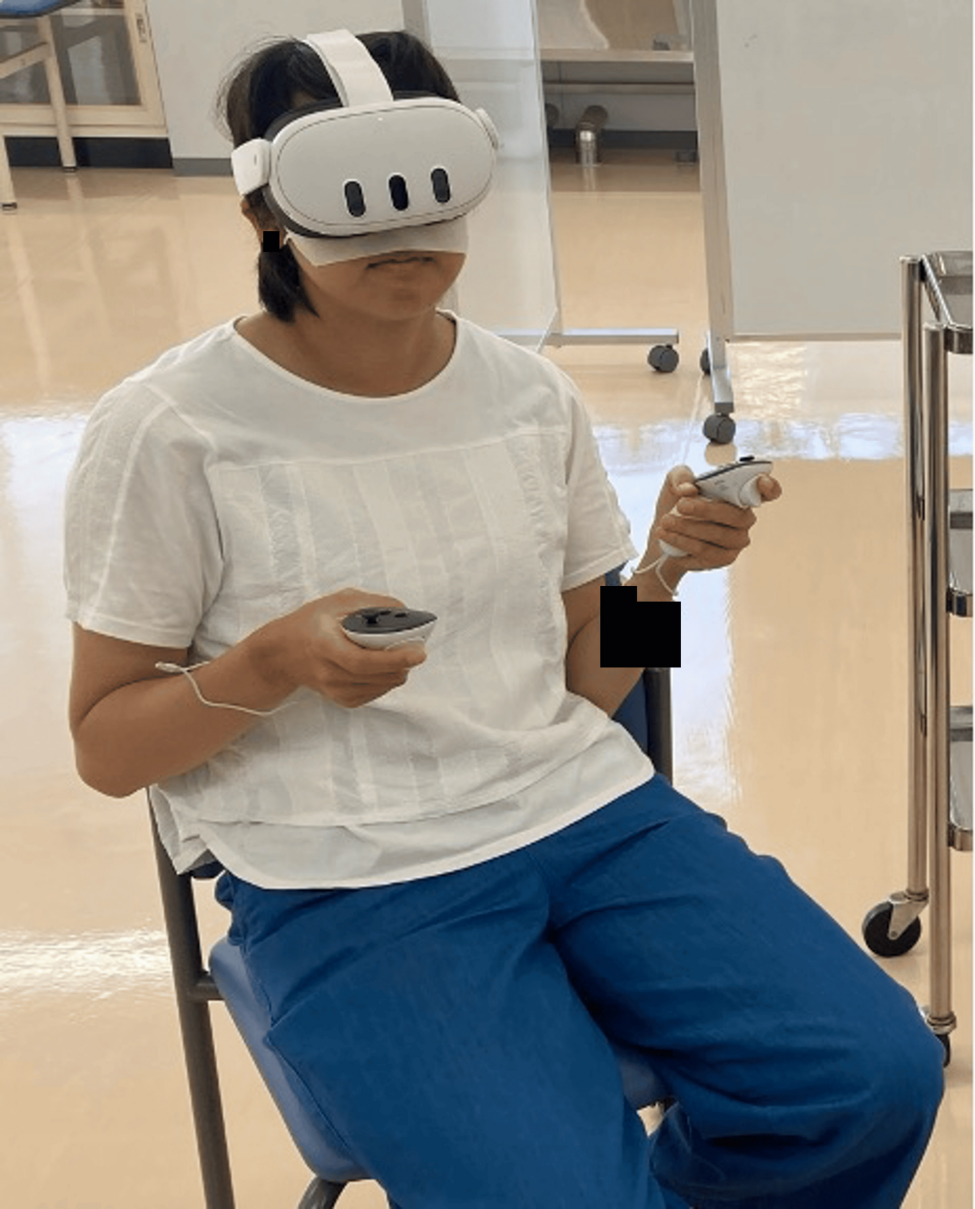
A participant wearing Meta Quest 3 The participants wore Meta Quest 3, created the avatars, and joined group discussions in Meta Horizon Workrooms.

The VR group used the MetaQuest 3 (Meta Platforms Inc., San Francisco, USA), which is a standalone wireless device that requires no connection to an external computer and is powered by internal batteries. The discussion software used was Meta Horizon Workrooms [12], which provides immersive VR spaces that enable users to communicate remotely through avatars (Figure 1). This avatar reflects the user’s voice, sound direction, facial orientation, arm movements, and mouth during speech. Furthermore, users can personalize their avatars by choosing from various hairstyles, clothing, and makeup options. The participants wore the Meta Quest 3, created their avatars, and participated in group discussions in the Meta Horizon Workrooms (Figure 2).

Video Group

Zoom (Zoom Video Communications Inc., San Francisco, USA) was used as the discussion software. Zoom is a videoconferencing tool that enables users to share their own views and voices via computers and tablet devices. The participants used their personal computers to join the group discussions in the Zoom meetings.

The discussion topic selected focused on the challenges faced by patients with chronic pain. Chronic pain is a subjective, multifactorial symptom, and its management requires the exchange of diverse opinions and collaborative decision-making [13], making it a suitable topic for evaluating discussion methods. All participants in both groups discussed the challenges experienced by patients with chronic shoulder pain, which is one of the most common symptoms according to the Comprehensive Survey of Living Conditions in Japan [14]. During the discussion, the participants were given two tasks: (1) listing 10 challenges in the patients’ daily lives caused by shoulder pain and (2) ranking the challenges in order of impact on their lives. The start and end of the discussion were determined by mutual agreement among all participating students in each group.

Data collection

To measure the quality of the discussion, team performance, and psychological distress associated with these, six data collection tools were selected based on previous research and reports. These tools were used to evaluate various aspects of the intervention and its outcomes. All the data collection tools were administered after the discussion sessions were completed.

Participant Characteristics

The first tool was a questionnaire designed to collect data on the participants’ characteristics. These included their age, sex, and prior experience with VR and Zoom meetings.

Discussion Implementation Metrics

The second measurement focused on the implementation of the discussion, which assessed the time taken for the discussions, side effects such as cybersickness [15], the number of participants who dropped out of the discussion, and the level of understanding of chronic shoulder pain. In previous studies, the average dropout rate for VR use was 16% [16]. In this study, VR discussion would be considered feasible if the completion rate of the discussions was ≥80%. The dropout rate was the main endpoint of the study to evaluate the feasibility of VR discussions. The level of understanding of chronic shoulder pain was rated on a 4.0-point Likert scale before and after the discussion, with higher scores indicating greater understanding.

Teamwork Self-Assessment Sheet

The third tool was a self-assessment sheet created by the Ministry of Economy, Trade, and Industry in Japan, which measured six elements required for teamwork [17]. These elements included opinion expression, listening, flexibility, situational awareness, discipline, and stress control. Each element was rated on a 5.0-point Likert scale, with higher scores indicating that these elements were demonstrated. The reliability and validity of this tool have not been verified.

Psychological Safety Scale

The fourth measurement assessed psychological safety using Edmondson’s Psychological Safety Scale [18], which has already been evaluated for reliability and validity. It consists of seven items that reflect the thoughts and feelings necessary for safe team activities. Each item was measured on a 7.0-point Likert scale (1 represents very inaccurate; 7 represents very accurate).

Cognitive Load Questionnaire

The fifth measurement assessed cognitive load using the naïve rating questionnaire [19], which has already been evaluated for reliability and validity. It consists of seven items that reflect the degree of engagement and mental resources directed toward the tasks. Each item was rated on a 7.0-point Likert scale (1 represents very inaccurate; 7 represents very accurate).

VR Discussion Impressions Questionnaire

The sixth tool was a questionnaire, administered only to the VR discussion group, to gather participants’ impressions of using VR and avatars. The questionnaire consisted of questions on the intention to use VR group discussions, external characteristics of the avatars created, the relevance of avatars and discussions, and a free-entry section for additional comments about the discussions.

Statistical analyses

Descriptive statistics were calculated for both the VR and video groups. All variables are summarized by group. Continuous variables are presented as means and standard deviations (SDs). Categorical variables are shown as medians, interquartile ranges, and frequencies and percentages, as appropriate. The Wilcoxon rank sum exact test was conducted to compare the discussion duration between the two groups. A p-value less than 0.05 was considered statistically significant. Statistical analyses were performed using R version 4.4.2 (R Foundation for Statistical Computing, Vienna, Austria).

## Results

A total of 36 nursing students participated in the study and were assigned to either the VR group (n=17) or the video group (n=19). All participants were female (100%), with a mean age of 20.56 (SD=0.65) years. While all participants had prior experience using Zoom meetings, only one participant in the VR group had prior experience using VR (Table 1).

**Table 1:** Participant characteristics SD: standard deviation; VR: virtual reality

Characteristic	VR group (n=17)	Video group (n=19)	Total (n=36)
Age (years), Mean (SD)	20.53 (0.80)	20.58 (0.51)	20.56 (0.65)
Female sex, n (%)	17 (100)	19 (100)	36 (100)
Experience, n (%)			
VR			
Yes	1 (5.8)	0	1 (2.7)
No	16 (94.1)	19 (100)	35 (97.2)
Zoom meetings			
Yes	17 (100)	19 (100)	36 (100)
No	0	0	0

The mean discussion duration was 603.0 (SD=104.98) seconds for the VR group and 558.4 (SD=89.32) seconds for the video group, with a difference of 44.6 (-112.0 to 196.0) seconds between the groups (p=0.547). None of the participants had cybersickness or dropped out of the discussion. However, the level of understanding of chronic shoulder pain was the same in both groups, both before and after the discussion at 3.0 (SD=1.0) (Table 2).

**Table 2:** Comparison of the discussion implementation metrics between VR and video groups CI: confidence interval; IQR: interquartile range; SD: standard deviation; VR: virtual reality ^1^Wilcoxon rank sum exact test. P-values indicate statistical comparisons between the VR group and the video group, with values less than 0.05 considered statistically significant.

Discussion implementation metrics	VR group (n=17)	Video group (n=19)	Difference (95% CI)	p-value^1^
Discussion time (s), Mean (SD)	603.0 (104.98)	558.4 (89.32)	44.6 (-112.0 to 196.0)	0.547
Side effects, n (%)	0	0	-	
Dropouts, n (%)	0	0		
Understanding of chronic shoulder pain, Median (IQR)				
before discussion	3.0 (1.0)	3.0 (1.0)		
after discussion	3.0 (1.0)	3.0 (1.0)		

Regarding the elements necessary for teamwork, all items in both groups were well-demonstrated, with scores of ≥4.0. Both groups scored 5.0 points, which is the highest score, for the item “Kept the rules of discussion and promises to the group members.” Only the VR group scored 5.0 points for the items “Understood the difference of opinions and the group members’ position” and “Understood the relationship to the group members and things” (Table 3). However, the reliability of this tool was evaluated using Cronbach’s alpha coefficient, and the result was α = 0.58.

**Table 3:** Comparison of the elements required for teamwork between the VR and video groups IQR: interquartile range; VR: virtual reality

Self-assessment sheet that measures six elements required for teamwork	VR group (n=17)	Video group (n=19)
Median (IQR)		
1. Conveyed opinions in a clear and understandable manner	4.0 (0)	4.0 (0)
2. Listened carefully to the group members' opinions	4.0 (1.0)	4.0 (1.0)
3. Understood the difference of opinions and the group members' position	5.0 (1.0)	4.0 (1.5)
4. Understood relationship to the group members and things	5.0 (1.0)	4.0 (1.0)
5. Kept the rules of discussion and promises to the group members	5.0 (1.0)	5.0 (0.5)
6. Corresponded to sources of stress	4.0 (1.0)	4.5 (1.5)

Regarding psychological safety, six of the seven items had equal scores between the groups. However, for the item “No one on this team would deliberately act in a way that undermines my efforts,” the VR group scored one point higher than the video group (Table 4).

**Table 4:** Comparison of the psychological safety point between the VR and video groups IQR: interquartile range; VR: virtual reality

Edmondson’s Psychological Safety Scale	VR group (n=17)	Video group (n=19)
Median (IQR)		
1. If you make a mistake on this team, it is often held against you.	2.0 (1.0)	2.0 (2.0)
2. Members of this team are able to bring up problems and tough issues.	5.0 (1.0)	5.0 (1.0)
3. Members of this team sometimes reject others for being different.	2.0 (1.0)	2.0 (1.5)
4. It is safe to take a risk on this team.	5.0 (1.0)	5.0 (2.5)
5. It is difficult to ask other members of this team for help.	3.0 (1.0)	3.0 (3.0)
6. No one on this team would deliberately act in a way that undermines my efforts.	7.0 (1.0)	6.0 (1.0)
7. Working with members of this team, my unique skills and talents are valued and utilized.	5.0 (1.0)	5.0 (1.0)

Regarding cognitive load, three of the seven items were rated equally by both groups. However, for the item ‘The design of this task was very inconvenient for learning,” the VR group scored one point higher than the video group. Conversely, in the VR group, the scores for the two items “My point while dealing with the task was to understand everything correctly” and “During the task, it was exhausting to find the important information” were one and two points lower, respectively, than those in the video group (Table 5).

**Table 5:** Comparison of the cognitive load point between the VR and video groups IQR: interquartile range; VR: virtual reality

Cognitive load measurement - the naive rating questionnaire	VR group (n=17)	Video group (n=19)
Median (IQR)		
1. For this task, many things needed to be kept in mind simultaneously.	5.0 (3.0)	5.0 (1.0)
2. This task was very complex.	3.0 (2.0)	3.0 (2.5)
3. I made an effort, not only to understand several details, but to understand the overall context.	6.0 (1.0)	6.0 (1.0)
4. My point while dealing with the task was to understand everything correct.	4.0 (3.0)	5.0 (1.0)
5. During the task, it was exhausting to find the important information.	3.0 (2.0)	5.0 (0.5)
6. The design of this task was very inconvenient for learning.	3.0 (2.0)	2.0 (3.0)
7. During this task it was difficult to recognize and link the crucial information.	3.0 (2.0)	3.0 (2.0)

**Figure 3:**
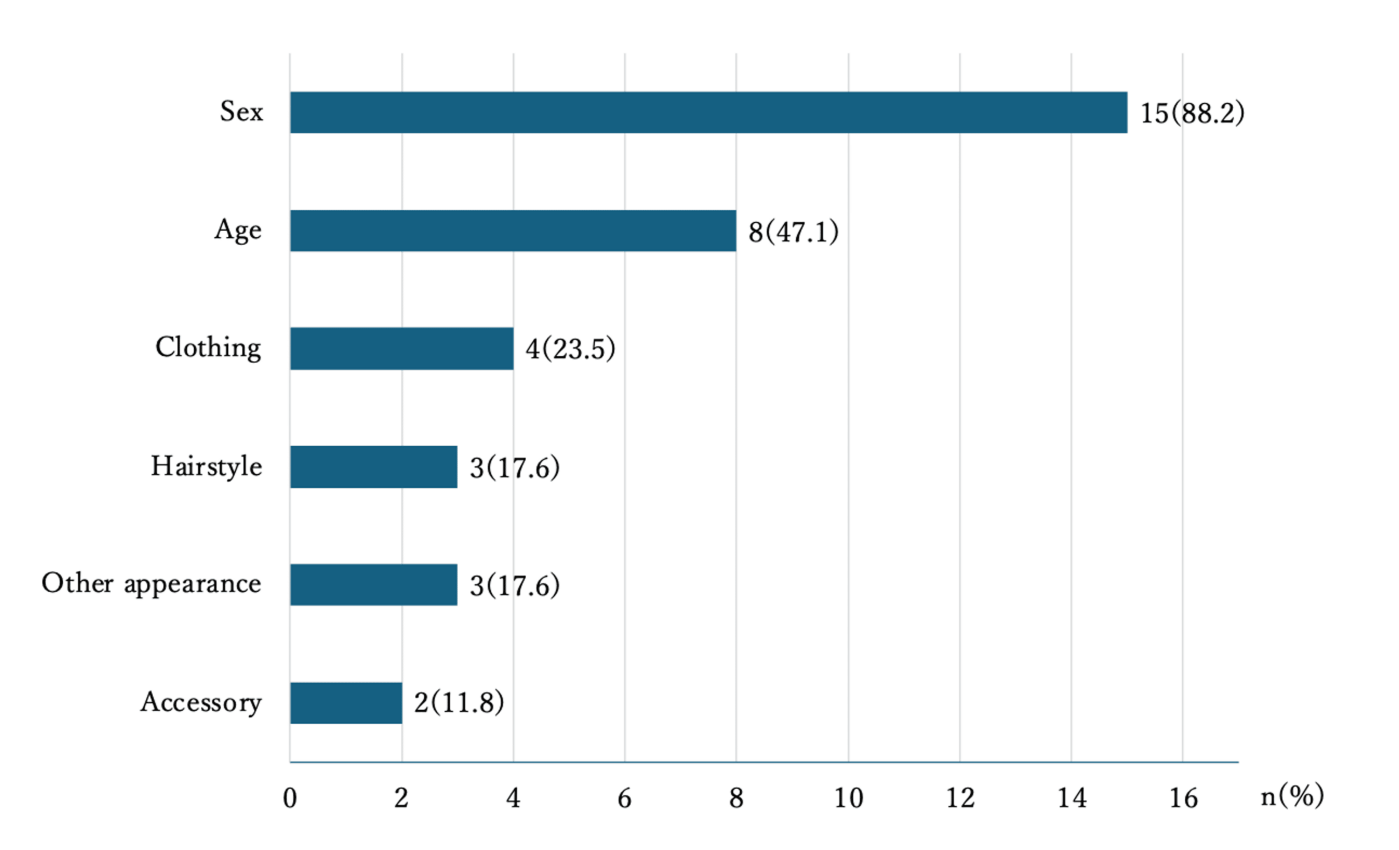
Characteristics of the avatars that reflect the participant students’ own in the VR group The participant students in VR group chose all applicable characteristics (n=17). VR: virtual reality

**Figure 4:**
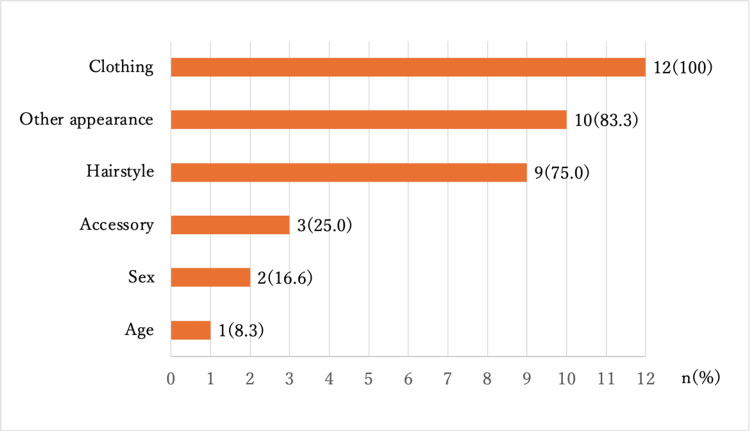
Characteristics of the avatars that influenced the discussions The participants in the VR group who answered that their avatars influenced the discussions chose all applicable characteristics (n=12). VR: virtual reality

All participants in the VR group expressed willingness to continue using VR for learning. Additionally, 12 (70.5%) participants indicated that avatar characteristics influenced the discussions (Table 6). Regarding the appearance of the avatar the participants created, most of them (88.2%) reflected their own “sex,” and approximately half (47.1%) reflected their own “age” (Figure 3). Regarding the avatar characteristics that influenced the discussion, all 12 students reported “clothing” (100%), and most of them selected “other appearance (excluding hairstyle, clothing, and accessories)” (83.3%) and “hairstyle” (75.0%) (Figure 4). The results of the free-entry questionnaire in the VR group included comments such as “I was able to have a discussion that was similar to the face-to-face discussion,” regarding the use of VR and “I felt a difference in the speaker’s attitude depending on their appearance, such as their clothes, hairstyle, and makeup,” regarding the use of avatars (Table 7).

**Table 6:** Discussion impressions of the use of VR and avatars in the VR group VR: virtual reality

VR discussion impressions questionnaire	VR group (n=17)
Intention to continue utilizing VR for learning, n (%)	
Yes	17 (100)
No	0
Influence of the avatar on discussion, n (%)	
Yes	12 (70.5)
No	5 (29.5)

**Table 7:** Results of free-entry questionnaire in the VR group VR: virtual reality

Participants impressions of using VR and avatars	VR group (n=17)
Regarding VR	Regarding avatar
I was able to have a discussion that was similar to the face-to-face discussion.	I've always been bad at speaking up, but I was able to speak up using the avatar.
I was able to hear the voices naturally and have a smooth conversation.	It was very fun to see the video moving in sync with my own movements, and to see my mouth move in sync with the video.
I felt that it takes some getting used to in order to continue for a long time.	I was able to have a fun discussion by creating my favorite avatar.
I thought it would be unsuitable for long discussions because it might get tiring.	I felt a difference in the speaker's attitude depending on their appearance, such as their clothes, hairstyle, and makeup.
Even though we were apart, I felt like I was sitting next to the members.	-
I have always been bad at speaking up, although I was able to speak up using an avatar.	-

## Discussion

This study investigated the feasibility and safety of online group discussions using VR, targeting nursing students, by comparing the VR and video groups. The following findings were clarified: (1) both groups engaged in discussions of similar quality and completed the discussion without experiencing any cybersickness and dropouts; (2) both groups demonstrated positive effects regarding teamwork elements and psychological safety; (3) the VR group had lower cognitive load than the video group on some items; and (4) in the VR group, all participants intended to continue utilizing VR for learning, and almost all participants recognized the influence of avatar characteristics on the discussion.

The mean discussion duration was approximately 10 minutes for both the VR and video groups, and no significant difference was observed in the level of understanding of chronic shoulder pain. All participants had prior experience using Zoom meetings, and almost all participants had no prior experience with VR. While VR can cause cybersickness to users after less than 10 minutes of virtual exposure [20], no cybersickness was observed in the participants, with no dropouts. Thus, VR group discussions for nursing students were considered feasible and safe. Notably, cybersickness increases with longer VR exposure [21], and in this study, the length of the discussion may have influenced the incidence of cybersickness and dropouts.

Both discussion groups had equally positive effects on teamwork elements and psychological safety. Regarding teamwork, two items were more positive in the VR group than in the video group: “Understood the difference of opinions and the group members’ positions” and “Understood the relationship between the group members and things.” In the VR group, communication via avatars facilitated the exchange of glances, use of gestures, and comprehension of each other’s locations [22], which may have increased their understanding of the opinions and relationships within the group. Regarding psychological safety, the following item was more positive in the VR group than in the video group: “No one on this team would deliberately act in a way that undermines my efforts.” In the video group, participants could only see the faces of the group members, which made it difficult to grasp important elements of communication, such as gestures [23]. This limitation may have affected their confidence in the absence of deliberate actions from others. However, regarding teamwork and psychological safety, no clear differences in effects were observed between the VR and video groups. This is likely because the participants knew each other, and a certain level of teamwork and psychological safety had already been established beforehand.

The VR group had a lower cognitive load than the video group on two items: “My point while dealing with the task was to understand everything correctly” and “During the task, it was exhausting to find the important information.” The high cognitive load of video conferencing may explain why the video group had difficulty focusing on the task [2]. In contrast, the VR group found it easier to understand the overall discussion, likely because the immersive virtual environment allowed them to focus better. However, no consensus exists on whether VR or video has a higher cognitive load, and no conclusions have been reached in the field of nursing education [24]. This study may have been influenced by factors such as discussion topics and tasks.

In the VR group, all participants intended to continue using VR for learning purposes. The free-entry questionnaire on the discussions using VR revealed several key opinions. One participant mentioned, “I was able to have a discussion that was similar to the face-to-face discussion,” while another stated, “Even though we were apart, I felt like I was sitting next to the members.” These responses align with the findings of a previous study that reported the social presence of VR fosters a sense of connection similar to face-to-face discussions [22]. Additionally, this study observed a lower cognitive load related to task focus compared to that in the video group. One participant noted, “I have always been bad at speaking up, although I was able to speak up using an avatar,” indicating that the anonymity provided by the avatar may have encouraged more participation than in face-to-face discussions [25].

Regarding avatar characteristics, most participants in the VR group reflected their age and sex. As for the avatar characteristics that influenced the discussions, almost all participants cited external characteristics, such as clothing and hairstyle. Although the participants did not specify how these characteristics affected the discussion, one response in the free-entry questionnaire highlighted, “I felt a difference in the speaker’s attitude depending on their appearance, such as their clothes, hairstyle, and makeup.” This suggests that the appearance of the avatar can influence both the user’s and others’ perceptions during the discussion [26].

Thus, the participants may have felt that they were influenced by the characteristics of their hairstyle, clothing, and makeup, as these are likely to reflect personality. Regarding age and sex, although the participants did not report being affected by these characteristics, they might have unconsciously felt more comfortable speaking, as in face-to-face discussions with members of the same generation and sex. In the medical field, studies have explored the effects of avatar body shape and sex on rehabilitation [27]. However, few studies have investigated the use of avatar functions in nursing education [24]. Adjusting avatar characteristics to align with learning objectives may be useful in nursing education.

This study clarified four main points. Research comparing VR and video conferencing in education has demonstrated that VR enhances communication and interaction among students [28,29], aligning with this study’s findings. Conversely, studies on the role of VR in communication within educational settings remain limited, highlighting the need for further research [30]. Further development is also necessary for this study.

Limitations

This study has some limitations. First, the participants were not randomly assigned to groups, which may have introduced selection bias. Second, the reliability of the teamwork self-assessment sheet used in this study was low (α=0.58), and the fact that the survey administrator and analyst were the same individuals may have introduced information bias. Last, the study was conducted at a single institution with a small sample size and only included female participants, which limits the generalizability of the findings.

## Conclusions

This study evaluated the feasibility and safety of online group discussions using VR and compared the effects of two online group discussion methods on nursing students. The absence of cybersickness and dropouts among the participants suggests that VR discussions are feasible and safe. However, no significant differences were observed in the participants’ understanding of the themes or evaluation items between the two methods. Future research should focus on refining the discussion themes, measurement scales, and avatar conditions to further validate the effectiveness of online discussion methods.
